# Desert Farming Benefits from Microbial Potential in Arid Soils and Promotes Diversity and Plant Health

**DOI:** 10.1371/journal.pone.0024452

**Published:** 2011-09-02

**Authors:** Martina Köberl, Henry Müller, Elshahat M. Ramadan, Gabriele Berg

**Affiliations:** 1 Institute for Environmental Biotechnology, Graz University of Technology, Graz, Austria; 2 Faculty of Agriculture, SEKEM and Heliopolis University, Cairo, Egypt; Argonne National Laboratory, United States of America

## Abstract

**Background:**

To convert deserts into arable, green landscapes is a global vision, and desert farming is a strong growing area of agriculture world-wide. However, its effect on diversity of soil microbial communities, which are responsible for important ecosystem services like plant health, is still not known.

**Methodology/Principal Findings:**

We studied the impact of long-term agriculture on desert soil in one of the most prominent examples for organic desert farming in Sekem (Egypt). Using a polyphasic methodological approach to analyse microbial communities in soil as well as associated with cultivated plants, drastic effects caused by 30 years of agriculture were detected. Analysing bacterial fingerprints, we found statistically significant differences between agricultural and native desert soil of about 60%. A pyrosequencing-based analysis of the 16S rRNA gene regions showed higher diversity in agricultural than in desert soil (Shannon diversity indices: 11.21/7.90), and displayed structural differences. The proportion of Firmicutes in field soil was significantly higher (37%) than in the desert (11%). *Bacillus* and *Paenibacillus* play the key role: they represented 96% of the antagonists towards phytopathogens, and identical 16S rRNA sequences in the amplicon library and for isolates were detected. The proportion of antagonistic strains was doubled in field in comparison to desert soil (21.6%/12.4%); disease-suppressive bacteria were especially enriched in plant roots. On the opposite, several extremophilic bacterial groups, e.g., *Acidimicrobium, Rubellimicrobium* and *Deinococcus-Thermus,* disappeared from soil after agricultural use. The N-fixing *Herbaspirillum* group only occurred in desert soil. Soil bacterial communities were strongly driven by the a-biotic factors water supply and pH.

**Conclusions/Significance:**

After long-term farming, a drastic shift in the bacterial communities in desert soil was observed. Bacterial communities in agricultural soil showed a higher diversity and a better ecosystem function for plant health but a loss of extremophilic bacteria. Interestingly, we detected that indigenous desert microorganisms promoted plant health in desert agro-ecosystems.

## Introduction

In contrast to desertification, which is recognised as a major threat to biodiversity, to convert deserts into arable, green landscapes is a global vision as well as competent answer to world hunger and climate change [1], [2]. Desert farming, which generally relies on irrigation, is one way to this vision. Agriculture systems were already developed in arid landscapes by ancient cultures, yet nowadays, there is a dramatically increasing need for large-scale desert farming to feed the population. For example, in Egypt, desert farmland is expected to grow about 40% till 2017, but this needs about five billion m^3^ of water a year [3]. These enormous amounts of water and the expected impact on the climate conditions are the major disadvantages of agriculture in the desert. While these problems are well-investigated, the effect on the bio-resource soil was yet not assessed. Moreover, there is still a gap of knowledge about the effect of management and land uses on the bacterial diversity of soils, which new molecular tools like metagenomics can help to close [4], [5].

Deserts represent extreme environments for microorganisms [6]. Although the conditions varied strongly in the different regions of the world, all of them are characterised by a combination of extreme temperatures and desiccation, high soil salinity, low nutrient levels, high summer UV radiation levels, and physical instability caused by strong winds: all factors contribute to the visual appearance of a sterile environment. While early studies supported this “sterility” by very low levels of viable/cultivable microorganisms, applications of new methods in microbial ecology led to interesting new findings and showed a contrasting picture [6], [7]. For example, in their global-scale study, Fierer & Jackson [8] found that the acidic soils of tropical forests harbour fewer bacterial taxa than the neutral pH soils of deserts. In McMurdo Dry Valleys, a hyperarid polar desert, microbial soil communities were relatively depauperate but harboured a broad range of previously unreported bacteria and fungi from polar regions [9]. In different sites in the Negev Desert, archaeal and bacterial diversity analysed by fingerprints using T-RFLP of the 16S rRNA genes was not constrained by precipitation, although the taxonomic composition differed [10]. In soil of the Atacama Desert, a high diversity of microorganisms known for life in hypersaline environments was found by analysis of DGGE profiles [11]. Most of the desert microbial communities seem to be structured solely by a-biotic processes [6], [7]. But, if adapted desert plants occurred, e.g. *Panicum* and *Stipagrostis* in Sinai or *Reaumuria negevensis* in Negev, they strongly shaped soil microbial diversity [12], [13]. However, all these investigations showed a unique and extraordinary microbial diversity in desert soils. An understanding of diversity in such microbial communities can be used to assess potential effects of desert farming on soil ecosystem services like plant health [14]. Emerging problems with soil-borne pathogens limited the plant yield after several years often drastically. Due to their specific ecology soil-borne pathogens are difficult to suppress; disease-suppressive bacteria, which are able to antagonise and biologically control them, provide a promising and sustainable solution [15].

The objective of this study was to analyse the effect of desert farming on soil microbial diversity and on disease-suppressive bacteria. We studied microbial diversity in native Egyptian desert soil in comparison to the agricultural soil, which was used more than 30 years for organic agriculture in Sekem farms (www.sekem.com; Egypt). To study the role of plant-associated bacteria in the agricultural soil, we analysed microbial communities in the rhizosphere and endorhiza of cultivated medical plants. Sekem is not only one of the most prominent examples of organic farming in the desert; they were assigned for social entrepreneurship [16], [17]. For this study, we used a broad set of methods including i) bacterial fingerprints using 16S rRNA PCR-SSCP (Single Strand Conformational Polymorphism) analysis to compare the communities at statistical level and identify the dominant bacterial taxa, ii) pyrosequencing-based 16S rRNA profiling to get a deeper insight into the soil communities, iii) a cultivation approach to assess the impact on disease-suppressive bacteria, and iv) a multivariate statistical analysis to identify environmental factors driving microbial communities. We demonstrate that long-term organic agriculture had a strong impact on microbial community structure and function, and identified highly specialised communities in all microenvironments.

## Results

### Molecular fingerprinting of microbial communities

**Figure 1:**
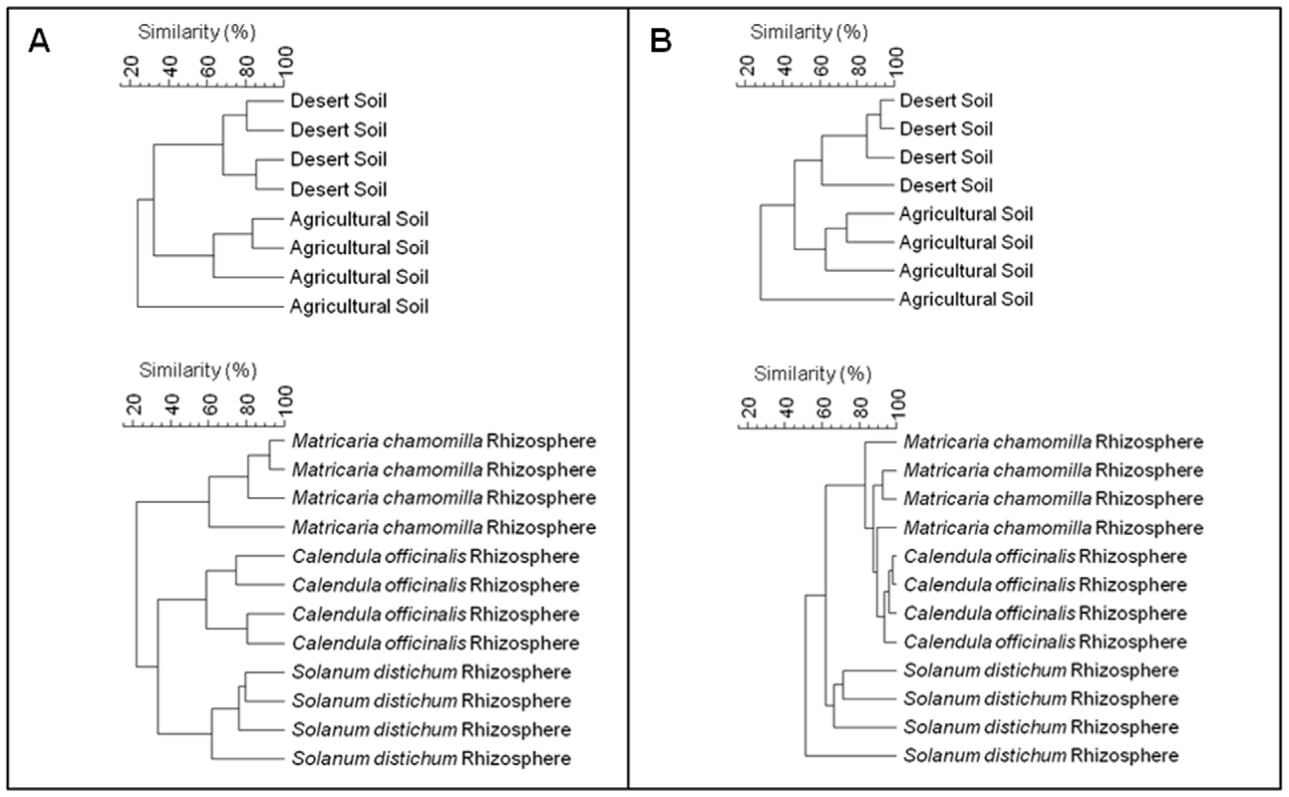
UPGMA dendrograms of total bacterial (A) and *Pseudomonas* (B) communities in soil and rhizosphere of the medical plants. The dendrograms were generated from the SSCP community profiles with GelCompar II. Following settings were used: dendrogram type: unweighted pair group method with arithmetic mean (UPGMA); similarity coefficient: curve based: Pearson correlation; position tolerances: optimisation: 4%, position tolerance: 1%.

To get a first overview about the structure of the bacterial communities, fingerprints were performed by SSCP analysis of 16S rRNA genes amplified from DNA obtained from desert and agricultural soil. In addition, we analysed bacterial communities from rhizosphere and endorhiza of the dominant plants German chamomile (*Matricaria chamomilla* L.), pot marigold (*Calendula officinalis* L.) and *Solanum distichum* Schumach. & Thonn. cultivated on farms. In comparison to the desert, in field soil an impressive diversity of bacteria was found (Fig. S1). According to cluster analyses, the composition of the bacterial community of agricultural soil differed significantly from the desert soil by approximately 60% of the bacterial strains (Fig. 1). In the bacterial community of desert soil two dominant bands could be detected, which were also abundant in all samples from the rhizosphere and endorhiza of all three investigated medical plants (Fig. S1). The two dominant bands were identified by partial 16S rRNA gene sequence analysis as *Ochrobactrum* sp. (closest database match *O. grignonense,* 99% similarity to NR_028901) and *Rhodococcus* sp. (closest database match *R. erythropolis*, 99% similarity to NR_037024). Further, *Bacillus* sp. was found nearly in all samples (closest database match *B. subtilis* subsp. *subtilis*, 99% similarity to NR_027552). For the rhizosphere as well as for the endorhiza of the medical plants a clear plant-specific effect of the bacterial communities was found (Fig. 1). They shared only 20% of the bacterial community, whereas the majority was determined by plant-specific bacteria.

### Pyrosequencing-based 16S rRNA profiling of the bacterial community in soil

**Figure 2:**
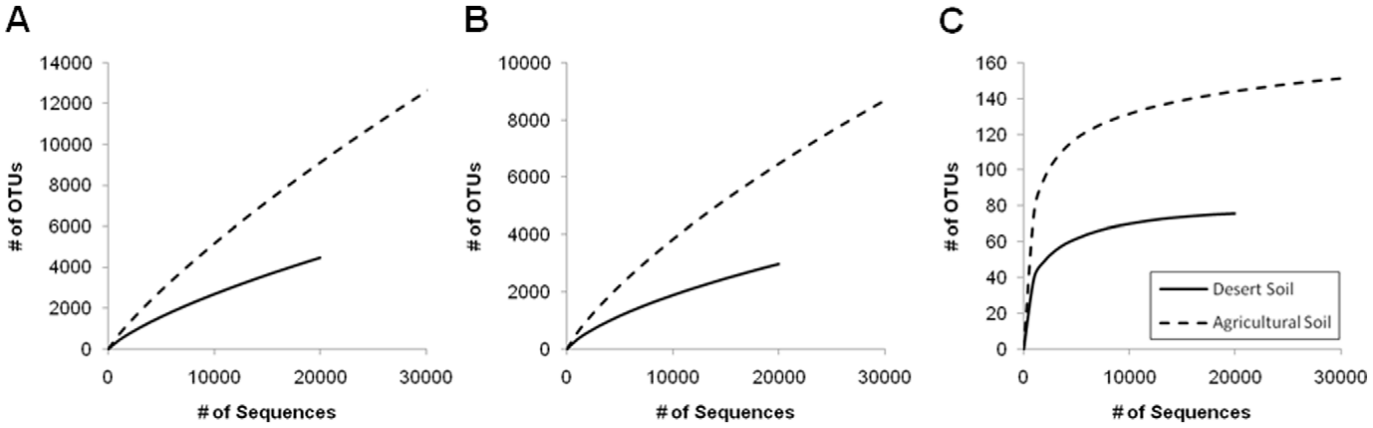
Rarefaction analyses of the two soil types. Rarefaction curves indicate that the diversity of bacterial phylotypes is higher in the agricultural soil compared to the surrounding desert soil. OTUs are shown at genetic distance levels of 3% (A), 5% (B) and 20% (C). Data were calculated by employing tools of the RDP pyrosequencing pipeline (http://pyro.cme.msu.edu).

To deeply survey the diversity and the composition of the bacterial communities present in untreated desert soil and after 30 years of organic agriculture, a pyrosequencing-based analysis of partial 16S rRNA gene sequences (V4-V5 region) has been employed. In desert soil, we recovered 19,244 and in agricultural soil 33,384 quality sequences with a read length of ≥ 150 bp. Of all quality sequences 83.0% could be classified below the domain level; this proportion is in accordance with other pyrosequencing-based studies [18]-[20]. To determine rarefaction curves, operational taxonomic units (OTUs) were identified at sequence divergences of 3% (species level), 5% (genus level) and 20% (phylum level). The rarefaction analysis of the bacterial community in the desert soil in comparison to the agricultural soil is shown in Fig. 2. At a dissimilarity level of 20%, both curves show a clear saturation. Thus the surveying effort covered almost the full extent of taxonomic diversity at this level of genetic distance. Additionally, a comparison of rarefaction analyses with the number of OTUs estimated by the Chao1 richness estimator [20] revealed that in both soils over 90% of the estimated taxonomic richness was covered by the sequencing effort (Table 1). At the genus level (5% dissimilarity) the full extent of taxonomic diversity was not surveyed (42% and 31%). The computed Shannon indices of diversity (H’) were much higher for the agricultural soil than for the desert soil, this indicates a higher bacterial diversity in soil due the agricultural use of the desert.

**Table 1:** Species richness estimates obtained at 3%, 5% and 20% genetic dissimilarity from pyrosequencing of 16S rRNA from metagenomic DNA extracted from desert soil and agricultural soil.

	Shannon index^a^ (H’)			Rarefaction^b^ (no. of OTUs)			Chao1^c^ (no. of OTUs)			Coverage (%)		
	3%	5%	20%	3%	5%	20%	3%	5%	20%	3%	5%	20%
Desert Soil	7.90	7.04	3.02	4,465	2,967	76	13,278	7,012	77	33.6	42.3	98.8
Agricultural Soil	11.21	9.94	3.91	9,112	6,474	144	38,985	20,838	161	23.4	31.1	90.0

a a higher number indicates more diversity;

b the results from the rarefaction analyses are also depicted in Fig. 2;

c nonparametric richness estimator based on the distribution of singletons and doubletons.

**Figure 3:**
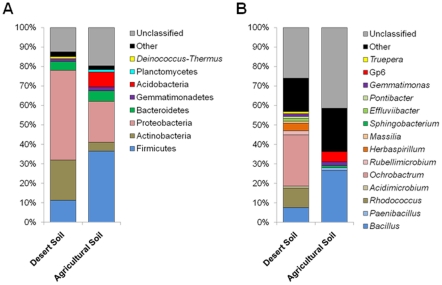
The bacterial communities in the two different soil types. Relative clone composition of major phyla (A) and genera (B) was determined by pyrosequencing of 16S rRNA from metagenomic DNA extracted from desert and agricultural soil. The identification of the closest strain based on 16S rRNA sequence similarity was achieved using the web server SnoWMAn 1.7 (http://snowman.genome.tugraz.at). Phylogenetic groups accounting for ≤1% of all quality sequences are summarised in the artificial group others.

The 43,673 classifiable sequences obtained from both soil types together were affiliated with 18 different phyla. Proteobacteria (30.2%), Firmicutes (27.3%) and Actinobacteria (10.5%) were the dominant phyla (Fig. 3, Table S1). These dominant phyla were present in both soils. In detail, Firmicutes are highly enriched in agricultural soil (from 11.3% in desert soil to 36.6% in agricultural soil), Proteobacteria (46.0% in desert soil and 21.0% in agricultural soil) and Actinobacteria (20.7% in desert soil and 4.6% in agricultural soil) occurred in farmland in lower concentrations than in the surrounding desert. Further, in both soils Bacteroidetes (4.6% and 5.3%) and Gemmatimonadetes (1.4% and 1.9%) were present. Considering only phyla covering more than 1% of quality sequences, Acidobacteria (7.9%) and Planctomycetes (1.1%) were only found in the agricultural soil, and *Deinococcus-Thermus* (1.1%) was only detectable in the desert sand. These abundances of the phyla coincided with results from previously reported meta-analysis of bacterial community composition in soils and, despite the special soil type of the desert, the composition covers rather well with studies of completely different soils [18], [20]-[22] with exception of Firmicutes. Most of the Firmicutes sequences were classified as belonging to the genus *Bacillus*; in the agricultural soil also *Paenibacillus* was found (5% of classified Firmicutes). In desert soil *Ochrobactrum* was the most abundant genus within the (Alpha-)Proteobacteria (79% of classified Proteobacteria) and *Rhodococcus* among the Actinobacteria (90% of classified Actinobacteria). The Acidobacteria in the agricultural soil are affiliated only with subdivision 6.

### Quantitative analysis of bacterial abundances

A cultivation-independent approach (total 16S rRNA gene copy numbers using quantitative PCR) was combined with cultivation, which was also the basis for isolate screening, to assess bacterial abundances in the different microhabitats. Copy numbers in agricultural soil were 9.4±0.5 log_10_ g^−1^ compared to 8.1±1.1 log_10_ g^−1^ in desert soil (Fig. S2), and were not statistically significantly different. In contrast, abundances of culturable bacteria determined on R2A resulted in statistically significant higher abundances in agricultural soil (7.7±0.4 log_10_ CFU g^−1^) in comparison to desert soil (4.6±0.6 log_10_ CFU g^−1^). The rhizosphere of all three investigated medical plants was highly colonised by culturable bacteria: log_10_ CFU ranged from 7.8±0.3 to 8.0±0.2 g^−1^ fw. In contrast, in the endorhiza significantly lower CFUs were detected with log_10_ 2.0±0.2 to 3.7±0.8 g^−1^ fw.

### Antagonistic potential of the bacterial community towards pathogenic fungi

A cultivation approach was used to analyse a functional aspect of the bacterial communities. To assess the indigenous anti-phytopathogenic potential, the antagonistic activity against three major soil-borne phytopathogenic fungi *Verticillium dahliae*, *Rhizoctonia solani* and *Fusarium culmorum* was determined. From each microenvironment up to 200 isolates were randomly selected and assessed regarding their anti-phytopathogenic capacity *in vitro*. All isolated soil bacteria (199 isolates from desert soil and 155 isolates from agricultural soil) were screened by dual testing regarding their antagonistic activity towards *V. dahliae*, *R. solani* and *F. culmorum* (Table 2). In general, bacterial isolates obtained from the soil of the farm exhibited a higher *in vitro* antagonistic potential towards soil-borne phytopathogenic fungi in comparison to the bacteria isolated from the desert soil (agricultural soil 21.6±0.8%; desert soil 12.4±0.7%). From the agricultural soil, 17.4% (27 isolates) demonstrated *in vitro* broad-spectrum antagonism towards all three pathogens, from the desert soil 10.6% (21 isolates) were able to suppress the growth of all fungi tested. No enrichment of antagonists in the rhizosphere and endorhiza of the investigated medical plants was detected. In general, *M. chamomilla* and *S. distichum* showed a higher antagonistic potential than *C. officinalis*. Especially the endorhiza from *M. chamomilla* harboured a high proportion of antagonists. Whereas in the soil and in the rhizosphere could be found most antagonistic bacteria towards *F. culmorum*, in the endorhiza of the medical plants most antagonists showed antagonism towards *V. dahliae*.

**Table 2:** Proportions of bacterial isolates antagonistic towards the soil-borne fungal pathogens *Verticillium dahliae*, *Rhizoctonia solani* and *Fusarium culmorum*.

		Proportion of antagonists (%)^a^		
Microhabitat	Origin	*V. dahliae*	*R. solani*	*F. culmorum*
Soil	Desert Soil	11.1±1.8	12.8±0.6	13.4±0.1
	Agricultural Soil	20.0±1.6	21.9±2.2	22.6±1.4
Rhizosphere	*Matricaria chamomilla*	12.5±2.9	8.3±0.7	13.0±1.8
	*Calendula officinalis*	9.0±0.5	7.1±0.1	10.1±3.1
	*Solanum distichum*	13.7±2.3	13.8±3.8	15.7±0.0
Endorhiza	*Matricaria chamomilla*	19.9±1.8	16.4±2.3	18.8±2.6
	*Calendula officinalis*	4.2±2.9	0.0±0.0	1.4±1.0
	*Solanum distichum*	13.5±5.1	10.4±5.8	12.5±5.8

a Data are averages of 1^st^ and 2^nd^ sampling ± confidences.

**Figure 4:**
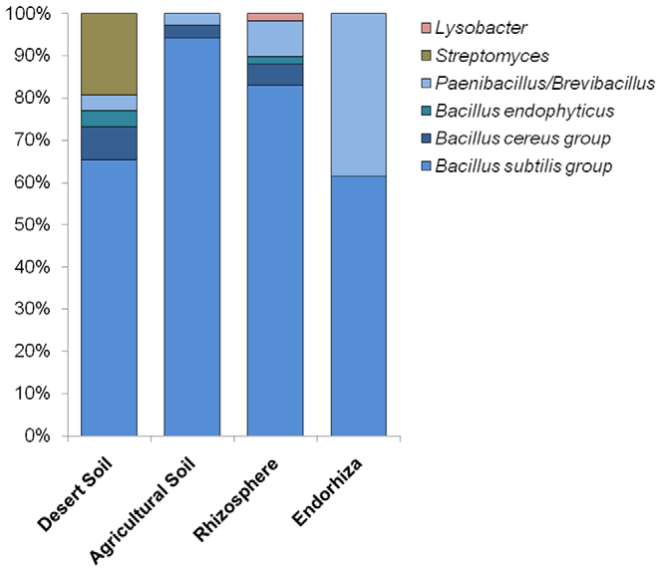
Diversity of bacterial antagonists with an activity towards pathogenic fungi. Isolates with activity against two pathogens were identified by partial 16S rRNA gene sequencing. Samples from rhizosphere and endorhiza include isolates from the medical plants *Matricaria chamomilla*, *Calendula officinalis* and *Solanum distichum*.

To assess the diversity of bacterial antagonists, isolates with an activity towards at least two of the soil-borne pathogenic fungi (162 isolates) were characterised genotypically and identified by partial 16S rRNA gene sequencing (Fig. 4). Using restriction fragment length polymorphism, of the 16S rRNA ( =  amplified ribosomal RNA gene restriction analysis [ARDRA]), the antagonistic isolates could be clustered into six groups: (1) *Bacillus subtilis*, (2) *Bacillus cereus*, (3) *Bacillus endophyticus*, (4) *Paenibacillus*/*Brevibacillus*, (5) *Streptomyces,* and (6) *Lysobacter*. With the exception of the *Lysobacter* strain (only one isolate from the rhizosphere of *M. chamomilla*, closest database match *L. enzymogenes*), only Gram-positive antagonists were found. All antagonistic populations were dominated by Firmicutes; *Bacillus* and *Paenibacillus* could be isolated from all habitats. Interestingly, antagonistic isolates of the genus *Streptomyces* were found exclusively in desert soil.

**Figure 5:**
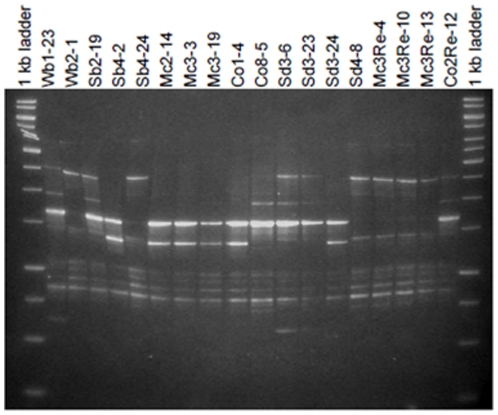
BOX PCR fingerprints of genetically very similar antagonists of the *Bacillus subtilis* group isolated from different microenvironments. The similarity in the dendrogram between them was more than 80%. Isolates were encoded by abbreviations: (1) soil type or plant species (Wb  =  desert soil; Sb  =  Sekem soil, Mc  =  *Matricaria chamomilla*, Co  =  *Calendula officinalis*, Sd  =  *Solanum distichum*), (2) replicate (1–4), (3) microenvironment (Re  =  endorhiza, rhizosphere and soil have no further designation), and (4) consecutive number of the isolate per replicate.

To analyse the genotypic diversity within the taxonomic groups at population level, BOX PCR patterns of the whole bacterial genome were used. Especially within the large *Bacillus subtilis* cluster (123 isolates), a high genotypic diversity was found. At a cut off level of 80%, they could be divided into 37 genotypic groups. By partial 16S rRNA gene sequencing isolates were identified as *B. subtilis* subsp. *subtilis* and *spizizenii*, *B. vallismortis*, *B. mojavensis* and *B. atrophaeus*. The *Paenibacillus*/*Brevibacillus* isolates could be divided into eight BOX clusters and *Bacillus endophyticus* into two. *Streptomyces* was subdivided in three genotypes, the closest database matches were *S. peucetius*, *S. scabiei* and *S. subrutilus*. Surprisingly, among the *Bacillus subtilis* group, isolates with identical BOX patterns could be detected in desert soil as well as in the agricultural soil, and also in rhizosphere and endorhiza of the medical plants (Fig. 5). Based on unique genotypic patterns and antagonistic potential, 45 promising biocontrol strains were selected of which 89% belonged to the Bacillales (Table S2).

### Ecological factors driving the bacterial communities in soil

Indirect correspondence analysis (CA) based on the OTUs of soil species obtained by microbial fingerprinting showed the coherence and similarity of the different samples indicated by crowding points at a CA biplot (data not shown). Furthermore, the influence of the environmental factors on the bacterial soil communities was examined using the multivariate statistical analysis. A significant effect was proved for water supply (precipitation + irrigation) (regression coefficient: 0.3760), pH (0.3719) and, to a lower extent for organic carbon (0.1600) and soil quality (0.1011).

## Discussion

Agriculture in deserts open new ways to solve diverse problems: produce enough food for poor regions e.g. in Africa, produce renewable crops for industrial applications and to capture and restore CO_2_ in soil. However, agricultural use induces a drastic shift for the whole ecosystem, and risk assessments to evaluate the function are necessary. Here we analysed differences of microbial communities in undisturbed desert soil in comparison to desert soil, which was cultivated under organic (biodynamic) conditions for 30 years. Altogether, a strong impact of long-term agriculture on microbial community structure and function was identified, which will be discussed and assessed in detail.

The composition of the bacterial communities in desert and agricultural soil differed strongly. In microbial fingerprints, both communities showed about 60% differences. Using a pyrosequencing-based approach of the 16S rRNA gene region, reasons for these differences could be identified. The relative abundance of Firmicutes in agricultural soil was significantly enhanced from 11 to 37%. Especially the proportion of 37% is remarkable because Janssen [22] reported them to contribute only a mean of 2% (range 0-8%) in the total bacterial soil community. *Bacillus* and *Paenibacillus* play the key role to explain this difference; they were dominant in the 16S rRNA gene amplicon library and represented 96% of the antagonists towards phytopathogens identified in the culturable fraction. In addition, by microbial fingerprints we showed that this Gram-positive group was enriched in the rhizosphere as well as endorhiza of medical plants cultivated in Sekem. Interestingly, we found *Bacillus* isolates with the same BOX pattern in desert and field soil as well as in the rhizosphere and endorhiza of medical plants, which was confirmed also by our bacterial fingerprint analysis. Furthermore, identical 16S rRNA gene sequences were found for isolates as well as in the amplicon library. This is further evidence for enrichment of plant rhizosphere-specific bacteria from the soil. Moreover, it indicates that the antagonistic bacteria were enriched from desert soil and not from the compost treatment. Both, *Bacillus* and *Paenibacillus* are well-characterised plant-associated genera with antagonistic properties towards fungal plant pathogens [23]. While the proportion of Firmicutes was enhanced in field soil, several extremophilic bacterial groups, e.g. *Acidimicrobium*, *Rubellimicrobium* and *Deinococcus-Thermus* disappeared. Bacteria from all of these genera/phyla are either impossible or else extremely difficult to cultivate and only found in extreme environments by molecular analysis. For example, bacteria from the phylum *Deinococcus-Thermus* possess important adaptations such as resistance to environmental hazards, e.g., desiccation, ultraviolet radiation, high salinity, and high temperatures [24]. In general, the proportion of cultivable bacteria was lower in desert soil than in field soil, which was shown in the comparison between results obtained by cultivation and qPCR analysis. Based on our pyrosequencing data, bacterial communities in agricultural soil were characterised by a higher diversity than in desert soil (Shannon diversity indices: agricultural soil 11.21; desert 7.90). The high bacterial diversity found in the organically managed soil was shown for agriculture in the desert for the first time but was already reported for another organically managed system [14].

Additionally, for the function of the bacterial communities in desert and agricultural soil we found strong differences. The proportion of strains with antagonistic *in vitro* activity against soil-borne phytopathogens was statistically significantly enhanced in agricultural soil in comparison to desert soil. Other current studies showed also that organic farming methods can mitigate ecological damages caused by pests and pathogens by promoting natural enemies, analysed for example in the pathosystem potato – potato beetle [25] or grape – *Botrytis cineria* [26]. Although the proportion of antagonistic strains was higher in agricultural soil, their diversity was much lower. All of the isolated antagonists belong to the *Bacillus/Paenibacillus* group. In contrast, in desert soil, diverse antagonistic *Streptomyces* species were identified, including *Streptomyces peucetius*, a species known to produce anthracycline antibiotics [27]. Another interesting fact is that members of the *Herbaspirillum* group, most of them known as N-fixing species, only occurred in desert soil. In most of the deserts, plants have a very short period to develop. This fact is well-known, when shortly after a rainfall millions of seedlings occur and colour the whole desert in green. Therefore, plants need plant growth promoting rhizobacteria, and *Herbaspirillum* strains belong to this group. Owing to compost treatment agricultural soils are saturated with nitrogen. These facts could explain that this important functional group had a lower abundance in field soil. Another genus with an interesting occurrence is *Orchrobactrum*. In desert soil *Ochrobactrum* was the most abundant genus within Proteobacteria and also in microbial fingerprints this genus was found in high abundance in soil but also in the rhizosphere/endorhiza of medical plants. Bacteria of this genus are known for its ambivalent interaction with eukaryotes, while they show plant growth promotion effects on plants, they can cause opportunistic infections in humans [28].

What are the reasons for the changes in structure and function of the bacterial community? The main factor, which explains the differences, is the continuous irrigation of farm land. This factor was identified by an indirect correspondence analysis. Precipitation in this arid region is general low (21-52 mm). The agriculture is completely dependent on irrigation water coming from the Nile or from local ground water. Irrigation systems were used to supply about 2,500-2,600 l m^-3^ per year. The aridity level was also one of the main factors that shaped the microbial community structure in patchy desert landscapes of Negev [29]. By the multivariate statistic, the pH of soil was identified as the second impact factor. This factor was often reported as main driver, e.g. in global studies of microbial communities in soil [8], [18]. Another factor, which contributed to the shift in the bacterial community in a lower extend, is compost treatment. This was already shown for other examples in organic agriculture: due to the use of compost, studies have found that biodynamic farms have a significantly better soil quality than conventionally farmed soils but comparable to the soil quality achieved by other organic methods [14], [17]. The compost treatment is responsible for nutrient and organic matter supply. On the other side, compost is known for an extremely high but also specific bacterial diversity. No evidence was found for an impact of these specifically adapted bacteria on soil communities. One factor, which could be not included in the statistical analyses, is plant-specific enrichment of bacterial communities. The extent of plant specificity was shown in a study of *Verticillium* host plants published by Smalla et al. [30], and later described for many other plant species [31]. In our study, we found a highly pronounced effect for each of the medical plants investigated. All three medical plants, which belong to the dominant herbs in Sekem and were included in the study, are known for their production of secondary metabolites. For example, German chamomile, for which we found the strongest effect, is used medicinally to treat sore stomach and irritable bowel syndrome. Chamomile plants produce the terpene bisabolol, and other active ingredients like farnesene, chamazulene, flavonoids and coumarin [32]. Some of them are known for their anti-microbial properties, and others, such as flavonoids often serve as signals in plant-microbe interactions [33].

In a final assessment, bacterial communities in agricultural soil showed a higher diversity and a better ecosystem function for plant health, which was measured as proportion of disease-suppressive bacteria. On the other side, there is a loss of extremophilic bacteria, which are typical inhabitants of desert soil. However, due to the fact that all farms are still surrounded by desert, we can conclude that also this specific diversity is maintained. The most interesting fact detected in our study was that indigenous desert microorganisms fulfil important functions in desert agro-ecosystems: *Bacillus* and *Paenibacillus* strains were enriched via plant roots from desert soil. This was shown at the population level using genotypic fingerprinting by BOX pattern, at community level by microbial fingerprints as well as in the metagenome.

## Materials and Methods

The experimental design comprise samples from agricultural soil, rhizosphere and endorhiza samples from main medical crops cultivated in Sekem farms as well as samples from the surrounding desert soil from two different sampling times. All sampling sites are private property of the Sekem companies. The sampling was done in cooperation with Angela Hoffmann and Elshahat M. Ramadan (Sekem) with permission of Ibrahim Abouleish, the owner of Sekem, for a joint project. Therefore, no other permit was required. Samples from agricultural used soil were taken at the Sekem farm Adleya, located in the North-eastern desert region of Egypt near Bilbeis (30°22′88"N; 31°39′41"E). The agriculture was completely dependent on irrigation water (2,607 l m^-3^ on average per year) coming from the Nile or from local ground water drillings; sprinkler and drip irrigation systems were used. The farmland soil was fertilised with compost that was produced on their own composting facility, where rice straw, water hyacinth, wood chips, organic waste, clay, chicken and cow manure was used as input materials. The compost was applied twice a year (May and September), during the preparation of the fields for the cropping season. The soil texture at the Sekem farm was classified by Luske & van der Kamp [17] as loamy sand (pH 8.4) with an organic carbon content of 0.8% and a clay content of 4%. Desert soil was collected from two sites in the surrounding desert uninfluenced by human activities (30°35'01"N; 32°25'49"E; 29°52′26″N, 31°13′1″E) and was classified as sand (pH 7.7) with an organic carbon content of <0.2% and a clay content of 1.5%. Desert soil was characterised by a low moisture level; plants were very scarce [17]. At each site, four composite samples of soil in a horizon of 10–30 cm depth were collected. Furthermore, from three different species of medical plants (German chamomile [*Matricaria chamomilla* L.], pot marigold [*Calendula officinalis* L.] and *Solanum distichum* Schumach. & Thonn.) planted on the Adleya farm (30°22′88"N; 31°39′41"E), roots with adhering soil were obtained. From each plant four independent composite samples consisted of 5–10 plants were taken. At the first sampling time (October 2009), *Matricaria chamomilla* and *Calendula officinalis* have been in the seedling stage, whereas the samples from the perennial *Solanum distichum* were taken from lignified plants. At the second sampling time (April 2010), all medical plants were in the flowering stage.

To isolate total community DNA from soil and from rhizosphere for all cultivation independent analyses, 5 g of soil/roots with adhering soil and 45 ml of 0.85% NaCl were mixed for 5 min on the vortex. For the isolation from the endorhiza, 5 g material of roots were surface-sterilised with 4% NaOCl for 5 min, then the roots were washed three times with sterile Aqua dest. After 10 ml sterile 0.85% NaCl were added the roots were homogenised using mortar and pestle. For isolation of total DNA from soil, rhizosphere and endorhiza 4 mL of the liquid parts were centrifuged at high speed (16,000×g, 4°C) for 20 min and resulting microbial pellets were stored at −70°C. In the desert soil, a lower concentration of DNA was expected. Therefore, for the isolation of total DNA the pellets of 10 ml supernatant were used. Total community DNA was extracted using the FastDNA® SPIN Kit for Soil (MP Biomedicals, Solon, USA) according to the manufacturer's protocol and used for fingerprints and the deep-sequencing approach.

Fingerprinting of microbial communities by Single Strand Conformational Polymorphism Analysis (SSCP) was carried out as described by Schwieger & Tebbe [34]. Bacterial 16S rRNA gene sequences were amplified by PCR using the eubacterial primer pair Unibac-II-515f (5′-GTG CCA GCA GCC GC-3′) and Unibac-II-927r^P^ (5′-CCC GTC AAT TYM TTT GAG TT-3′) [35]. The PCR was performed by using a total volume of 60 µl containing 1 × Taq&Go (MP Biomedicals, Eschwege, Germany), 1.5 mM MgCl_2_, 0.2 µM of each primer and 1 µl of template DNA (95°C, 5 min; 32 cycles of 95°C, 20 s; 54°C, 15 s; 72°C, 30 s; and elongation at 72°C, 10 min). For the analysis of the *Pseudomonas* community a nested PCR was performed. In a first PCR *Pseudomonas* were selectively amplified with primers F311Ps (5′-CTG GTC TGA GAG GAT GAT CAG T-3′) and 1459rPs^P^ (5′-AAT CAC TCC GTG GTA AAC GT-3′) [36] followed by a second PCR with the primer pair Unibac-II-515f/Unibac-II-927r^P^. The reaction mixture for the first PCR (20 µl) was composed of 1 × Taq&Go, 2.25 mM MgCl_2_, 0.5 mg/ml BSA, 1.5% DMSO, 0.2 µM of each primer and 1 µl of template DNA (94°C, 7 min; 30 cycles of 94°C, 45 s; 56°C, 2 min; 72°C, 2 min; and elongation at 72°C, 10 min). Samples served as templates for the second PCR. The obtained amplicons were separated using the TGGE Maxi system (Biometra, Göttingen, Germany) at 400 V and 26°C. Silver staining was used for the routine detection of DNA bands in SSCP gels [37]. Dominant bands were excised from SSCP gels as described by Schwieger and Tebbe [34]. Extracted DNA fragments were re-amplified by PCR and sequenced. For phylogenetic analysis and identification of related sequences, the obtained sequences were aligned with reference gene sequences from GenBank using BLAST algorithm.

Computer-assisted comparisons of SSCP generated community profiles were performed by using the software GelCompar II (Applied Maths, Kortrijk, Belgium). The cluster analysis was performed using following settings: dendrogram type: unweighted pair group method with arithmetic mean (UPGMA); similarity coefficient: curve based: Pearson correlation; position tolerances: optimisation: 4%, position tolerance: 1% [38]. Furthermore, correspondence analysis was used to answer the question whether a correlation exists (1) between the independently sampled microbial communities of the different sampling points and (2) between soil communities and environmental factors. The following environmental data were used: i) soil quality (sand, loam, semi-loam), ii) soil pH, iii) content of organic carbon and iv) water supply (sum of local precipitation per year [21, 52 mm] and irrigation). According to the distance of the bands, the SSCP gels were theoretically divided into operational taxonomic units (OTUs). The presence or absence of individual amplified product DNA bands in each group was scored. The obtained matrix was used to compare data statistically using the indirect correspondence analysis for unimodal data of the software package Canoco 4.5 [39].

To analyse the taxonomic composition of the soil bacterial community by a deep-sequencing approach, the hypervariable V4-V5 region of the 16S rRNA gene (*Escherichia coli* positions 515 to 927) was amplified in a nested PCR approach for pyrosequencing. In a first PCR the primer pair 27f/1492r [40] was used and in the second PCR V4-V5 region was amplified with the following primer set, containing the 454 pyrosequencing adaptors and sample specific tags (underlined): Unibac-II-515f_MID13 (5′-CGT ATC GCC TCC CTC GCG CCA TCA GCA TAG TAG TG GTG CCA GCA GCC GC-3′) respectively Unibac-II-515f_MID14 (5′-CGT ATC GCC TCC CTC GCG CCA TCA GCG AGA GAT AC GTG CCA GCA GCC GC-3′) and Unibac-II-927r_MID13-14 (5′-CTA TGC GCC TTG CCA GCC CGC TCA G CCC GTC AAT TYM TTT GAG TT-3′). The reaction mixture for the first PCR (20 µl) contained 1 × Taq&Go, 0.25 µM of each primer and 1 µl of template DNA (95°C, 5 min; 30 cycles of 95°C, 30 s; 57°C, 30 s; 72°C, 90 s; and elongation at 72°C, 5 min). The second PCR was performed by using 1 × Taq&Go, 1.5 mM MgCl_2_, 0.4 µM of each primer and 2 µl of template DNA (95°C, 5 min; 32 cycles of 95°C, 20 s; 54°C, 15 s; 72°C, 30 s; and elongation at 72°C, 10 min). PCR products of four independent soil samples of the same habitat were pooled in equal volumes and purified by employing the Wizard® SV Gel and PCR Clean-Up System (Promega, Madison, USA). A total of 130 ng of amplified 16S rRNA gene product from each soil was required to construct the libraries for 454 pyrosequencing. For taxonomy-based analysis, the web server SnoWMAn 1.7 (http://snowman.genome.tugraz.at) [41] was employed. Sequences that were shorter than 150 bp in length or of low quality were removed from the pyrosequencing-derived data sets and following settings were used: analysis type: BLAT pipeline; reference database: greengenes_24-Mar-2010; rarefaction method: MOTHUR; taxonomy: RDP; confidence threshold: 80%; include taxa covering more than: 1%. For rarefaction analysis and ascertainment of diversity indices, the data were normalised considering the same number of sequences to all samples using default settings in the open source software package QIIME (http://qiime.sourceforge.net), which allows analysis of high-throughput community sequencing data [42]. Rarefaction curves were calculated by using the tools aligner, complete linkage clustering and rarefaction of the ribosomal database project (RDP) pyrosequencing pipeline (http://pyro.cme.msu.edu) [43]. Shannon [44] and Chao1 [45] indices were calculated based on the complete linkage clustering data.

The same region of the 16S rRNA gene was amplified by quantitative PCR to determine the total bacterial abundances in desert and agricultural soil. Reactions were conducted in a total volume of 10 µl containing 1 × KAPA^TM^ SYBR® FAST qPCR MasterMix Universal (PEQLAB, Polling, Austria), 0.25 µM of each primer (Unibac-II-515f and Unibac-II-927r [35]) and 1 µl template DNA (95°C, 5 min; 35 cycles of 95°C, 20 s; 54°C, 15 s; 72°C, 30 s; and melt from 72 to 95°C). Rotor-Gene^TM^ 6000 real-time rotary analyser (Corbett Research, Sydney, Australia) was used for quantification of fluorescence. For absolute quantification the PCR amplified 16S rRNA gene fragment was cloned into a pGEM®-T Easy Vector (Promega, Mannheim, Germany). Serial dilutions of PCR fragments generated with the primers usp (5‘-GTAAAACGACGGCCAGT-3′) and rsp (5′-CAGGAAACAGCTATGACC-3′), which specifically bind to sides flanking the multi cloning side of the Vector, were used as standard for calculation of copy number. Concentrations determined by absolute quantification were calculated to copy number per g soil. Each replicate was analysed three times in two independent runs. Significances in the difference between desert and agricultural soil were calculated using the independent samples *t* test with PASW Statistics 18 (SPSS Inc., Chicago, USA).

Same cell suspensions as used for the isolation of total community DNA were used for isolation of bacteria from soil, rhizosphere and endorhiza: They were used for dilution and plating on R2A (Roth, Karlsruhe, Germany) in duplicates. Plates were incubated for 4 days at room temperature (RT) and colony forming units were counted to calculate the means of colonies (log_10_ CFU) based on fresh weight (fw). If possible, for each replicate 24 bacterial isolates were selected and subcultured on nutrient agar (NA). The isolates were purified and then stored at −70°C in nutrient broth (NB) (Sifin, Berlin, Germany) containing 15% glycerol. Isolates were encoded using a combination of letters and numbers indicating: (1) soil type or plant species (Wb  =  desert soil; Sb  =  Sekem soil, Mc  =  *Matricaria chamomilla*, Co  =  *Calendula officinalis*, Sd  =  *Solanum distichum*), (2) replicate (1–4), (3) microenvironment (Re  =  endorhiza, rhizosphere and soil have no further designation), and (4) consecutive number of the isolate per replicate.

Altogether, 1,212 selected bacterial isolates were screened in dual-culture *in vitro* assays on Waksman agar (WA) [46] for their antagonistic potential towards *Verticillium dahliae* Kleb. V25, *Rhizoctonia solani* Kühn AG4, and *Fusarium culmorum* (Wm. G. Sm.) Sacc. E1. For *R. solani* and *F. culmorum* agar disks of 5 mm diameter with mycelia were directly cut out from PDA plates (Roth, Karlsruhe, Germany) and placed between the streaks of four bacterial isolates. *V. dahliae* was grown in liquid culture in Czapek Dox broth (Duchefa, Haarlem, Netherlands) at 20°C. 200 µl of the suspension containing hyphal fragments were plated onto the agar and after surface drying the isolates were placed on the same plate. Inhibition zones were measured after 4–7 days of incubation at RT. Each isolate was tested twice independently. From antagonistic isolates, DNA was prepared following the protocol of Berg et al. [46]. Amplified ribosomal RNA gene restriction analysis (ARDRA) using the restriction endonucleases *Hha*I (MP Biomedicals, Eschwege, Germany) and *Pst*I (New England Biolabs, Ipswich, UK) was used to group isolates at genus level. Isolates displaying similar ARDRA patterns were further analysed using BOX-PCR genomic fingerprinting. BOX-PCR fingerprints were performed using the BOX_A1R primer (5'-CTA CGG CAA GGC GAC GCT GAC G-3') as described by Rademaker and de Bruijn [47]. PCR conditions were used as specified by Berg et al. [28] and PCR products were separated by gel electrophoresis on 1.5% agarose gels. Antagonists with either individual ARDRA patterns or different BOX patterns (cut-off level 80%) were identified by partial 16S rRNA gene sequence analysis according to Berg et al. [46]. PCR product was sequenced with the Applied Biosystems 3130l Genetic Analyser sequencer, Data Collection v3.0, Sequencing Analysis v5.2 (Foster City, USA) at the sequencing core facility ZMF, Medical University of Graz, Austria. Obtained sequences were aligned with reference gene sequences from GenBank using BLAST algorithm. Sequences obtained were submitted to EMBL Nucleotide Sequence Database under accession numbers FR854236-FR854290.

## Supporting Information

Figure S1 **16S rRNA PCR-SSCP profiles of the bacterial communities in soil and endorhiza of the medical plants.** Std.: 1 kb DNA ladder. The following bands were identified as: 1. *Ochrobactrum grignonense,* 99% similarity to NR_028901 and 2. *Rhodococcus erythropolis* 99% similarity to NR_037024.
(TIF)

Figure S2 **Abundances of (A) total and (B) culturable bacteria in desert and agricultural soil.** Data for total bacteria were ascertained by qPCR of the 16S rRNA genes and data for culturable bacteria by isolation on R2A. Averages of 16S rRNA gene copy numbers and viable counts per gram soil as log_10_ and confidences are shown.
(TIF)

Table S1 **Relative composition of bacterial phyla, classes, orders, families and genera in desert and agricultural soil.**
(DOC)

Table S2 **Identification of selected bacterial antagonists isolated from different habitats.**
(DOC)

## References

[1] Clery D (2011). Environmental technology. Greenhouse-power plant hybrid set to make Jordan's desert bloom.. Science.

[2] Pereira AC, Gama VF (2010). Anthropization on the Cerrado biome in the Brazilian Uruçuí-Una Ecological Station estimated from orbital images.. Braz J Biol.

[3] Reuters (2007). Egypt plan to green Sahara desert stirs controversy.. Reuters website.

[4] Barot S, Blouin M, Fontaine S, Jouquet P, Lata JC (2007). A tale of four stories: soil ecology, theory, evolution and the publication system.. PLoS One.

[5] Acosta-Martínez V, Dowd S, Sun Y, Allen V (2008). Tag-encoded pyrosequencing analysis of bacterial diversity in a single soil type as affected by management and land use.. Soil Biol Biochem.

[6] Cary SC, McDonald IR, Barrett JE, Cowan DA (2010). On the rocks: the microbiology of Antarctic Dry Valley soils.. Nat Rev Microbiol.

[7] Caruso T, Chan Y, Lacap DC, Lau MC, McKay CP (2011). Stochastic and deterministic processes interact in the assembly of desert microbial communities on a global scale.. ISME J: In press.

[8] Fierer N, Jackson RB (2006). The diversity and biogeography of soil bacterial communities.. Proc Natl Acad Sci USA.

[9] Pointing SB, Chan Y, Lacap DC, Lau MC, Jurgens JA (2009). Highly specialised microbial diversity in hyper-arid polar desert.. Proc Natl Acad Sci USA.

[10] Angel R, Soares MI, Ungar ED, Gillor O (2010). Biogeography of soil archaea and bacteria along a steep precipitation gradient.. ISME J.

[11] de Los Ríos A, Valea S, Ascaso C, Davila A, Kastovsky J (2010). Comparative analysis of the microbial communities inhabiting halite evaporites of the Atacama Desert.. Int Microbiol.

[12] Othman AA, Amer WM, Fayez M, Hegazi NA (2004). Rhizosheath of Sinai desert plants is a potential repository for associative diazotrophs.. Microbiol Res.

[13] Saul-Tcherkas V, Steinberger Y (2011). Soil microbial diversity in the vicinity of a Negev Desert shrub – *Reaumuria negevensis*.. Microb Ecol.

[14] Mäder P, Fliessbach A, Dubois D, Gunst L, Fried P (2002). Soil fertility and biodiversity in organic farming.. Science.

[15] Mendes R, Kruijt M, de Bruijn I, Dekkers E, van der Voort M (2011). Deciphering the rhizosphere microbiome for disease-suppressive bacteria.. Science.

[16] Schieffer A, Lessem R (2009). Beyond social and private enterprise: towards the integrated enterprise.. Transit Stud Rev.

[17] Luske B, van der Kamp J (2009). Carbon sequestration potential of reclaimed desert soils in Egypt. Louis Bolk Instituut & Soil and More International.. Soil and More International website.

[18] Lauber CL, Hamady M, Knight R, Fierer N (2009). Pyrosequencing-based assessment of soil pH as a predictor of soil bacterial community structure at the continental scale.. Appl Environ Microbiol.

[19] Lazarevic V, Whiteson K, Huse S, Hernandez D, Farinelli L (2009). Metagenomic study of the oral microbiota by Illumina high-throughput sequencing.. J Microbiol Methods.

[20] Will C, Thürmer A, Wollherr A, Nacke H, Herold N (2010). Horizon-specific bacterial community composition of German grassland soils, as revealed by pyrosequencing-based analysis of 16S rRNA genes.. Appl Environ Micobiol.

[21] Hansel CM, Fendorf S, Jardine PM, Francis CA (2008). Changes in bacterial and archaeal community structure and functional diversity along a geochemically variable soil profile.. Appl Environ Microbiol.

[22] Janssen PH (2006). Identifying the dominant soil bacterial taxa in libraries of 16S rRNA and 16S rRNA genes.. Appl Environ Microbiol.

[23] Emmert EAB, Handelsman J (1999). Biocontrol of plant diseases: a (Gram-) positive perspective.. FEMS Microbiol Lett.

[24] Battistuzzi FU, Hedges SB (2008). A major clade of prokaryotes with ancient adaptations to life on land.. Mol Biol Evol.

[25] Crowder DW, Northfield TD, Strand MR, Snyder WE (2010). Organic agriculture promotes evenness and natural pest control.. Nature.

[26] Schmid F, Moser G, Müller H, Berg G (2011). Functional and structural microbial diversity in organic and conventional viticulture: organic farming benefits natural biocontrol agents.. Appl Environ Micobiol.

[27] Niraula NP, Kim SH, Sohng JK, Kim ES (2010). Biotechnological doxorubicin production: pathway and regulation engineering of strains for enhanced production.. Appl Microbiol Biotechnol.

[28] Berg G, Eberl L, Hartmann A (2005). The rhizosphere as a reservoir for opportunistic human pathogenic bacteria.. Environ Microbiol.

[29] Ben-David EA, Zaady E, Sher Y, Nejidat A (2011). Assessment of the spatial distribution of soil microbial communities in patchy arid and semi-arid landscapes of the Negev Desert using combined PLFA and DGGE analyses.. FEMS Microbiol Ecol.

[30] Smalla K, Wieland G, Buchner A, Zock A, Parzy J (2001). Bulk and rhizosphere soil bacterial communities studied by denaturing gradient gel electrophoresis: plant-dependent enrichment and seasonal shifts revealed.. Appl Environ Microbiol.

[31] Berg G, Smalla K (2009). Plant species and soil type cooperatively shape the structure and function of microbial communities in the rhizosphere.. FEMS Microbiol Ecol.

[32] McKay DL, Blumberg JB (2006). A review of the bioactivity and potential health benefits of chamomile tea (*Matricaria recutita* L.).. Phytother Res.

[33] Shaw LJ, Morris P, Hooker JE (2006). Perception and modification of plant flavonoid signals by rhizosphere microorganisms.. Environ Microbiol.

[34] Schwieger F, Tebbe CC (1998). A new approach to utilize PCR-single-strand-conformation polymorphism for 16S rRNA gene-based microbial community analysis.. Appl Environ Microbiol.

[35] Zachow C, Tilcher R, Berg G (2008). Sugar beet-associated bacterial and fungal communities show a high indigenous antagonistic potential against plant pathogens.. Microb Ecol.

[36] Milling A, Smalla K, Maidl FX, Schloter M, Munch JC (2004). Effects of transgenic potatoes with an altered starch composition on the diversity of soil and rhizosphere bacteria and fungi.. Plant Soil.

[37] Bassam BJ, Caetano-Anolles G, Gresshoff PM (1991). Fast and sensitive silver staining of DNA in polyacrylamid gels.. Anal Biochem.

[38] Kropf S, Heuer H, Grüning M, Smalla K (2004). Significance test for comparing complex microbial community fingerprints using pairwise similarity measures.. J Microbiol Methods.

[39] Leps J, Smilauer P (2003). Multivariate analysis of ecological data using Canoco..

[40] Lane DJ, Stackebrandt E, Goodfellow M (1991). 16S/23S rRNA sequencing.. Nucleic acid techniques in bacterial systematics.

[41] Stocker S, Snajder R, Rainer J, Trajanoski S, Gorkiewicz G (2011). SnoWMAn: High-throughput phylotyping, analysis and comparison of microbial communities..

[42] Caporaso JG, Kuczynski J, Stombaugh J, Bittinger K, Bushman FD (2010). QIIME allows analysis of high-throughput community sequencing data.. Nat Methods.

[43] Cole JR, Wang Q, Cardenas E, Fish J, Chai B (2009). The ribosomal database project: improved alignments and new tools for rRNA analysis.. Nucleic Acids Res.

[44] Shannon CE (1997). The mathematical theory of communication. 1963.. MD Comput.

[45] Chao A, Bunge J (2002). Estimating the number of species in a stochastic abundance model.. Biometrics.

[46] Berg G, Roskot N, Steidle A, Eberl L, Zock A (2002). Plant-dependent genotypic and phenotypic diversity of antagonistic rhizobacteria isolated from different *Verticillium* host plants.. Appl Environ Microbiol.

[47] Rademaker JLW, de Bruijn FJ, Caetano-Anollés G, Gresshoff PM (1997). Characterization and classification of microbes by rep-PCR genomic fingerprinting and computer-assisted pattern analysis.. DNA markers: protocols, applications and overviews.

