# Supplementary figures and images for: Desert Farming Benefits from Microbial Potential in Arid Soils and Promotes Diversity and Plant Health
Source: PLoS One. 2011 Sep 2;6(9):e24452. doi: 10.1371/journal.pone.0024452 (PMC3166316; doi:10.1371/journal.pone.0024452)

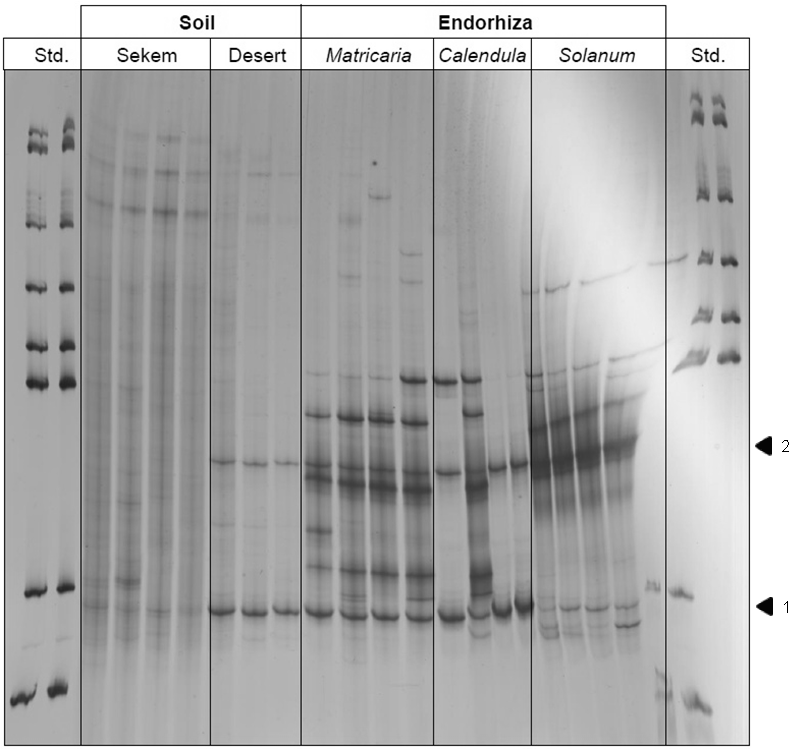

Supplement: Figure S1 — 16S rRNA PCR-SSCP profiles of the bacterial communities in soil and endorhiza of the medical plants. Std.: 1 kb DNA ladder. The following bands were identified as: 1. Ochrobactrum grignonense, 99% similarity to NR_028901 and 2. Rhodococcus erythropolis 99% similarity to NR_037024. (TIF) [file pone.0024452.s001.tif]

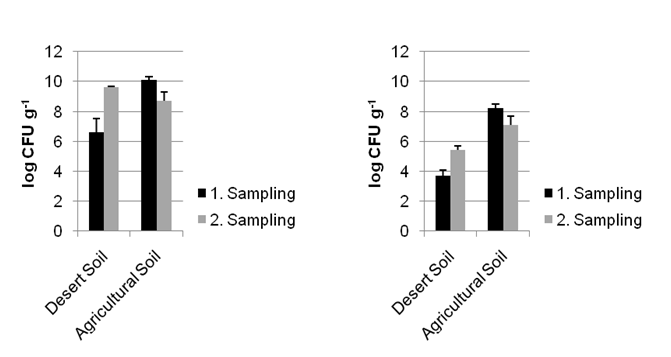

Supplement: Figure S2 — Abundances of (A) total and (B) culturable bacteria in desert and agricultural soil. Data for total bacteria were ascertained by qPCR of the 16S rRNA genes and data for culturable bacteria by isolation on R2A. Averages of 16S rRNA gene copy numbers and viable counts per gram soil as log10 and confidences are shown. (TIF) [file pone.0024452.s002.tif]
