# Supplementary material for: Desert Farming Benefits from Microbial Potential in Arid Soils and Promotes Diversity and Plant Health
Source: PLoS One. 2011 Sep 2;6(9):e24452. doi: 10.1371/journal.pone.0024452 (PMC3166316; doi:10.1371/journal.pone.0024452)
Supplement: Table S1 — Relative composition of bacterial phyla, classes, orders, families and genera in desert and agricultural soil. (DOC) [file pone.0024452.s003.doc]

**Table S1.** Relative composition of bacterial phyla. classes. orders. families and genera in desert and agricultural soil.

|  | Desert Soil^a^ | Agricultural Soil^a^ |
| --- | --- | --- |
| Phylum Level | | |
| Firmicutes | 11.3% | 36.6% |
| Actinobacteria | 20.7% | 4.6% |
| Proteobacteria | 46.0% | 21.0% |
| Bacteroidetes | 4.6% | 5.3% |
| Gemmatimonadetes | 1.4% | 1.9% |
| Acidobacteria |  | 7.9% |
| Planctomycetes |  | 1.1% |
| Deinococcus-Thermus | 1.1% |  |
| Other | 2.5% | 2.0% |
| Unclassified | 12.4% | 19.7% |
| Class Level | | |
| Bacilli | 10.9% | 35.4% |
| Actinobacteria | 20.7% | 4.6% |
| Alphaproteobacteria | 34.3% | 6.7% |
| Betaproteobacteria | 8.9% | 6.7% |
| Gammaproteobacteria | 1.6% | 3.1% |
| Deltaproteobacteria | 1.2% | 4.2% |
| Sphingobacteria | 3.3% | 2.9% |
| Flavobacteria |  | 2.0% |
| Bacteroidetes | 1.0% |  |
| Gemmatimonadetes | 1.4% | 1.9% |
| Acidobacteria |  | 7.9% |
| Planctomycetacia |  | 1.1% |
| Deinococci | 1.1% |  |
| Other | 2.9% | 3.0% |
| Unclassified | 12.8% | 20.6% |
| Order Level | | |
| Bacillales | 10.7% | 35.2% |
| Actinomycetales | 16.2% | 2.3% |
| Rubrobacterales | 2.0% |  |
| Acidimicrobiales | 1.1% |  |
| Rhizobiales | 29.5% | 4.3% |
| Rhodospirillales | 1.3% | 1.1% |
| Rhodobacterales | 2.5% |  |
| Burkholderiales | 8.4% | 3.0% |
| Oceanospirillales |  | 1.0% |
| Myxococcales |  | 1.1% |
| Sphingobacteriales | 3.3% | 2.9% |
| Flavobacteriales |  | 2.0% |
| Bacteroidales | 1.0% |  |
| Acidobacteriales |  | 7.9% |
| Gemmatimonadales | 1.4% | 1.9% |
| Planctomycetales |  | 1.1% |
| Deinococcales | 1.1% |  |
| Other | 6.8% | 7.6% |
| Unclassified | 14.8% | 28.7% |
| Family Level | | |
| Bacillales | 10.7% | 35.2% |
| Actinomycetales | 16.2% | 2.3% |
| Rubrobacterales | 2.0% |  |
| Acidimicrobiales | 1.1% |  |
| Rhizobiales | 29.5% | 4.3% |
| Rhodospirillales | 1.3% | 1.1% |
| Rhodobacterales | 2.5% |  |
| Burkholderiales | 8.4% | 3.0% |
| Oceanospirillales |  | 1.0% |
| Myxococcales |  | 1.1% |
| Sphingobacteriales | 3.3% | 2.9% |
| Flavobacteriales |  | 2.0% |
| Bacteroidales | 1.0% |  |
| Acidobacteriales |  | 7.9% |
| Gemmatimonadales | 1.4% | 1.9% |
| Planctomycetales |  | 1.1% |
| Deinococcales | 1.1% |  |
| Other | 6.8% | 7.6% |
| Unclassified | 14.8% | 28.7% |
| Genus Level | | |
| *Bacillus* | 7.6% | 26.6% |
| *Paenibacillus* |  | 1.4% |
| *Rhodococcus* | 9.9% |  |
| *Acidimicrobium* | 1.1% |  |
| *Ochrobactrum* | 26.4% |  |
| *Rubellimicrobium* | 2.1% |  |
| *Herbaspirillum* | 3.8% |  |
| *Massilia* | 1.0% |  |
| *Sphingobacterium* |  | 1.2% |
| *Effluviibacter* | 1.3% |  |
| *Pontibacter* | 1.0% |  |
| *Gemmatimonas* | 1.4% | 1.9% |
| Gp6 |  | 5.3% |
| *Truepera* | 1.1% |  |
| Other | 17.2% | 22.3% |
| Unclassified | 26.0% | 41.4% |

^a^determined by pyrosequencing of 16S rRNA from metagenomic DNA by using SnoWMAn 1.7.
