# Supplementary material for: Desert Farming Benefits from Microbial Potential in Arid Soils and Promotes Diversity and Plant Health
Source: PLoS One. 2011 Sep 2;6(9):e24452. doi: 10.1371/journal.pone.0024452 (PMC3166316; doi:10.1371/journal.pone.0024452)
Supplement: Table S2 — Identification of selected bacterial antagonists isolated from different habitats. (DOC) [file pone.0024452.s004.doc]

**Table S2. Identification of selected bacterial antagonists isolated from different habitats.**

| ARDRA group^a^ | Isolate  number | Microhabitat^b^ | Closest database match^c^ | Accession number | Similarity  (%) |
| --- | --- | --- | --- | --- | --- |
| A | Wb2n-1 | Desert Soil | *Bacillus vallismortis* | NR_024696 | 99% |
| A | Sb1-6 | Agricultural Soil | *Bacillus subtilis* subsp. *subtilis* | NR_027552 | 99% |
| A | Sb3-5 | Agricultural Soil | *Bacillus subtilis* subsp. *subtilis* | NR_027552 | 99% |
| A | Sb3-13 | Agricultural Soil | *Bacillus atrophaeus* | NR_024689 | 99% |
| A | Sb3-21 | Agricultural Soil | *Bacillus subtilis* subsp. *spizizenii* | NR_024931 | 99% |
| A | Sb3-24 | Agricultural Soil | *Bacillus subtilis* subsp. *subtilis* | NR_027552 | 99% |
| A | Sb4-14 | Agricultural Soil | *Bacillus vallismortis* | NR_024696 | 99% |
| A | Sb4-23 | Agricultural Soil | *Bacillus subtilis* subsp. *subtilis* | NR_027552 | 99% |
| A | Mc3-4 | Mc Rhizosphere | *Bacillus mojavensis* | NR_024693 | 98% |
| A | Mc5-18 | Mc Rhizosphere | *Bacillus subtilis* subsp. *subtilis* | NR_027552 | 99% |
| A | Mc5-19 | Mc Rhizosphere | *Bacillus subtilis* subsp. *subtilis* | NR_027552 | 99% |
| A | Co1-6 | Co Rhizosphere | *Bacillus subtilis* subsp. *subtilis* | NR_027552 | 99% |
| A | Co2-14 | Co Rhizosphere | *Bacillus subtilis* subsp. *spizizenii* | NR_024931 | 99% |
| A | Co7-19 | Co Rhizosphere | *Bacillus subtilis* subsp. *spizizenii* | NR_024931 | 100% |
| A | Sd1-14 | Sd Rhizosphere | *Bacillus subtilis* subsp. *spizizenii* | NR_024931 | 99% |
| A | Sd3-12 | Sd Rhizosphere | *Bacillus subtilis* subsp. *subtilis* | NR_027552 | 100% |
| A | Sd3-21 | Sd Rhizosphere | *Bacillus subtilis* subsp. *spizizenii* | NR_024931 | 99% |
| A | Sd7-15 | Sd Rhizosphere | *Bacillus subtilis* subsp. *spizizenii* | NR_024931 | 100% |
| A | Mc1Re-3 | Mc Endorhiza | *Bacillus subtilis* subsp. *subtilis* | NR_027552 | 99% |
| A | Mc2Re-2 | Mc Endorhiza | *Bacillus subtilis* subsp. *spizizenii* | NR_024931 | 99% |
| A | Mc2Re-9 | Mc Endorhiza | *Bacillus subtilis* subsp. *subtilis* | NR_027552 | 99% |
| A | Mc2Re-18 | Mc Endorhiza | *Bacillus subtilis* subsp. *subtilis* | NR_027552 | 99% |
| A | Mc2Re-21 | Mc Endorhiza | *Bacillus subtilis* subsp. *subtilis* | NR_027552 | 99% |
| A | Mc3Re-13 | Mc Endorhiza | *Bacillus subtilis* subsp. *subtilis* | NR_027552 | 98% |
| A | Mc5Re-2 | Mc Endorhiza | *Bacillus subtilis* subsp. *spizizenii* | NR_024931 | 100% |
| A | Mc5Re-15 | Mc Endorhiza | *Bacillus subtilis* subsp. *subtilis* | NR_027552 | 99% |
| A | Sd2Re-10 | Sd Endorhiza | *Bacillus mojavensis* | NR_024693 | 100% |
| A | Sd8Re-6 | Sd Endorhiza | *Bacillus subtilis* subsp. *spizizenii* | NR_024931 | 100% |
| A | Sd8Re-7 | Sd Endorhiza | *Bacillus subtilis* subsp. *subtilis* | NR_027552 | 99% |
| A | Sd8Re-23 | Sd Endorhiza | *Bacillus subtilis* subsp. *spizizenii* | NR_024931 | 100% |
| C | Wb1-13 | Desert Soil | *Bacillus endophyticus* | NR_025122 | 99% |
| C | Mc4-18 | Mc Rhizosphere | *Bacillus endophyticus* | NR_025122 | 99% |
| D | Wb2-3 | Desert Soil | *Paenibacillus polymyxa* | NR_037006 | 99% |
| D | Sb3-1 | Agricultural Soil | *Paenibacillus kribbensis* | NR_025169 | 99% |
| D | Mc2-9 | Mc Rhizosphere | *Paenibacillus brasilensis* | NR_025106 | 99% |
| D | Mc5-5 | Mc Rhizosphere | *Paenibacillus brasilensis* | NR_025106 | 99% |
| D | Mc6-4 | Mc Rhizosphere | *Brevibacillus limnophilus* | NR_024822 | 99% |
| D | Mc2Re-16 | Mc Endorhiza | *Paenibacillus brasilensis* | NR_025106 | 98% |
| D | Mc5Re-14 | Mc Endorhiza | *Paenibacillus polymyxa* | NR_037006 | 99% |
| D | Sd5Re-24 | Sd Endorhiza | *Paenibacillus brasilensis* | NR_025106 | 99% |
| E | Wb1n-4 | Desert Soil | *Streptomyces scabiei* | NR_025865 | 98% |
| E | Wb2n-2 | Desert Soil | *Streptomyces peucetius* | NR_024763 | 98% |
| E | Wb2n-11 | Desert Soil | *Streptomyces subrutilus* | NR_026203 | 99% |
| E | Wb2n-23 | Desert Soil | *Streptomyces peucetius* | NR_024763 | 98% |
| F | Mc1-3 | Mc Rhizosphere | *Lysobacter enzymogenes* | NR_036925 | 99% |

^a^the letters represent the different amplified rRNA gene restriction analysis patterns (A-F). group B (*Bacillus cereus* group) was completely excluded; ^b^Mc…*Matricaria chamomilla*. Co…*Calendula officinalis*. Sd…*Solanum distichum*; ^c^according to 16S rRNA gene sequencing.
